# *Candida albicans* orf19.3727 encodes phytase activity and is essential for human tissue damage

**DOI:** 10.1371/journal.pone.0189219

**Published:** 2017-12-07

**Authors:** Paul Wai-Kei Tsang, Wing-Ping Fong, Lakshman Perera Samaranayake

**Affiliations:** 1 Technological and Higher Education Institute of Hong Kong, HKSAR, China; 2 School of Life Sciences, The Chinese University of Hong Kong, HKSAR, China; 3 College of Dental Medicine, University of Sharjah, University City, United Arab Emirates; Louisiana State University, UNITED STATES

## Abstract

*Candida albicans* is a clinically important human fungal pathogen. We previously identified the presence of cell-associated phytase activity in *C*. *albicans*. Here, we reveal for the first time, that orf19.3727 contributes to phytase activity in *C*. *albicans* and ultimately to its virulence potency. Compared with its wild type counterpart, disruption of *C*. *albicans* orf19.3727 led to decreased phytase activity, reduced ability to form hyphae, attenuated *in vitro* adhesion, and reduced ability to penetrate human epithelium, which are the major virulence attributes of this yeast. Thus, orf19.3727 of *C*. *albicans* plays a key role in fungal pathogenesis. Further, our data uncover a putative novel strategy for anti-Candidal drug design through inhibition of phytase activity of this common pathogen.

## Introduction

The opportunistic human fungal pathogen *Candida albicans* poses significant clinical challenges causing recalcitrant infections both in community dwelling and hospitalized individuals. It is a frequent causative agent of recurrent, deep-seated and systemic lethal infections (candidiasis) in individuals with a suppressed immune system [1,2]. For instance, candidiasis is the fourth leading cause of hospital-acquired bloodstream infections, and disseminated candidiasis is associated with high morbidity and high mortality despite antifungal interventions [3,4].

Inorganic phosphate (Pi) and *myo*-inositol are essential nutrients for this yeast. Pi is a major component of adenosine triphosphate (ATP), a building block of the genetic materials (nucleic acids), and it is involved in many biochemical processes such as protein phosphorylation and glycolysis. *Myo*-inositol plays key roles in membrane formation, signal transduction, and osmoregulation [5]. Particularly, it is a precursor of the cell surface glycosylphosphatidylinositol(GPI)-anchored glycolipids which contribute to *C*. *albicans* virulence as GPI-anchored glycolipids interact with human macrophages during infection [6]. Thus, *myo*-inositol is critical for invasive Candidal infections whilst Pi metabolism has also been associated with virulence [7].

*Myo*-inositol and Pi are released from phytate hydrolysis by the action of phytase. Phytate, a major storage form of phosphorus in plants, is abundant in the human diet and intestine [5]. Phytase activity has been characterized in bacteria and fungi, with correlation to virulence in phytopathogens, and intestinal bacteria of the Atlantic cod [8–10]. Early studies have demonstrated phytase activity in *Candida* [8], and a Pi starvation induced phytase has been purified and functionally characterized from *C*. *krusei* [11].

Another remarkable virulence trait of *C*. *albicans* is its capability to switch between a budding yeast phase, and a filamentous phase (with pseudohyphae and true hyphae). This yeast-to-hyphal morphological transition, modulated by a variety of environmental cues and intracellular signalling pathways, is critical for tissue invasion. *C*. *albicans* strains defective in hyphal development are non-pathogenic [12–14].

Previously, we demonstrated the presence of cell-associated phytase activity in *Candida* [15]. Bioinformatics analysis revealed that *C*. *albicans* orf19.3727 shared high similarity to other yeast phytases. We generated *C*. *albicans* orf19.3727 null mutants by disrupting both chromosomal copies using a PCR-based gene targeting method, and investigated the functional role of orf19.3727 in yeast-to-hyphal morphogenesis, adhesion, and *in vitro* virulence. Our collective data suggest that *C*. *albicans* orf19.3727 contributes to phytase activity and is crucial for hyphal growth, adhesion, and cellular invasion, and ultimately to its pathogenic potential.

## Materials and methods

### Strains, cultivation and chemicals

All *C*. *albicans* strains and derivatives used in this study are listed in Table 1. They were obtained from the archival collection of the Oral Biosciences Laboratory of the Faculty of Dentistry, The University of Hong Kong. Fungal strains were routinely cultured in YPD medium (1% yeast extract, 2% peptone, 2% dextrose) at 30°C. Bactoagar (2%) was added in solid media. Fungal transformants were selected on minimal (0.67% yeast nitrogen base w/o amino acids, 2% dextrose) agar plates supplemented with uridine (50 Δg/mL) and/or histidine (50 Δg/mL). *Escherichia coli* DH5α (Novagen) was used for plasmid propagation and grown in LB medium (0.5% yeast extract, 1% tryptone, 0.5% NaCl) at 37°C. Other chemicals were obtained from commercial suppliers with the highest grade available.

**Table 1:** Fungal strains and primers used in this study.

Strains	Parent	Relevant genotypes
SC5314	-	Wild type
BWP17	SC5314	*ura3*::*λimm434*/*ura3*::*λimm434 arg4*::*hisG*/*arg4*::*hisG his1*::*hisG*/*his1*::*hisG*
BWP17 + CIp30	BWP17	*ura3*::*λimm434*/*ura3*::*λimm434 arg4*::*hisG*/*arg4*::*hisG his1*::*hisG*/*his1*::*hisG* CIp30::*URA3*/*HIS1*/*ARG4*
orf19.3727 null mutants	BWP17	*ura3*::*λimm434*/*ura3*::*λimm434 arg4*::*hisG*/*arg4*::*hisG his1*::*hisG*/*his1*::*hisG pho112*::*HIS1*/*pho112*::*ARG4*/CIp10::*URA3*
orf19.3727 reintegrants	orf19.3727 null mutants	*ura3*::*λimm434*/*ura3*::*λimm434 arg4*::*hisG*/*arg4*::*hisG his1*::*hisG*/*his1*::*hisG pho112*::*HIS1*/*pho112*::*ARG4*/CIp10::*PHO112*::*URA3*
Primers		5’ to 3’ sequence*
orf19.3727-ARG4F		tttttctactatgtgttttccatccattcccgttttttctttttgtttatttgtttatacatatatatatacatttaatttttaataattgttatttgtGAAGCTTCGTACGCTGCAGGTC
orf19.3727-ARG4R		gttggaaattttaaatttgtcagaaagtaaaaatgaactcagtttgaatttagtcactgatctcctgctgtcagaacttgcccttggtatatttgttgaaTCTGATATCATCGATGAATTCGAG
orf19.3727-HIS1F		tttttctactatgtgttttccatccattcccgttttttctttttgtttatttgtttatacatatatatatacatttaatttttaataattgttatttgtCGCGGGATCCTGGAGGATG
orf19.3727-HIS1R		gttggaaattttaaatttgtcagaaagtaaaaatgaactcagtttgaatttagtcactgatctcctgctgtcagaacttgcccttggtatatttgttgaaAAGGAAGTTTAAACGAGAATGCCTATTGAC
X2-CaARG4		AATGGATCAGTGGACCGGTG
X3-CaARG4		GAGGAGTACGACCTCAAGCGC
X2-CaHIS1		CAACGAAATGGCCTCCCCTACCACAG
X3-CaHIS1		GGACGAATTGAAGAAAGCTGGTGCAACCG

*For long primers, lower case sequences are homology region to orf19.3727, while upper case sequences anneal to pFA modules.

### Creation of *C*. *albicans* orf19.3727 null mutants and reintegrants

Genomic DNA of *C*. *albicans* SC5314 was isolated using a glass bead method [16]. Gene disruption cassettes for *C*. *albicans* orf19.3727 were constructed by PCR using gene-specific primers (Table 1) and plasmids pFA-ARG4 or pFA-HIS1 [17]. Transformation of *C*. *albicans* BWP17 (*ura3*, *his1*, *arg4*) with the gene disruption cassettes was performed using lithium acetate [18]. After the first round of gene deletion, heterozygous transformants were selected on minimal plates containing uridine and histidine at 30°C. Six transformants were randomly selected and verified using PCR with verification primers (X2-CaARG4/X3-CaARG4) (Table 1). For the second round deletion, heterozygous transformants were transformed with gene disruption cassettes derived from pFA-HIS1. Homozygous transformants were scored on minimal plates containing uridine. Six transformants were randomly selected and their genotypes were confirmed using PCR with verification primers (X2-CaHIS1/X3-CaHIS1) (Table 1) and genomic Southern hybridization. Two independent *C*. *albicans* orf19.3727 null mutants were randomly selected. The *ura3* auxotrophy in the null mutants was recovered by integrating plasmid CIp10 (carries *URA3*) into the *RPS10* locus. *C*. *albicans* BWP17 + CIp30 was created to serve as a control by transforming plasmid CIp30 (carries *URA3*, *HIS1*, *ARG4*) into *C*. *albicans* BWP17 at *RPS10* locus [18,19].

*C*. *albicans* orf19.3727 reintegrants were created by transforming full length orf19.3727 into the null mutants. The full length orf19.3727 was obtained by PCR using gene-specific primers (Table 1), and cloned into plasmid CIp10. The CIp10-orf19.3727 plasmid was transformed into the null mutants, and six transformants were randomly selected and verified by PCR.

### Determination of phytase activity

Cell-associated phytase activity was determined by measuring the amount of released Pi from sodium phytate [20]. The amount of liberated Pi was quantified by an ammonium molybdate method. One unit of phytase activity was defined as the amount of enzyme that yields 1 μmol of Pi per min. The amount of protein was quantified by BCA Protein Assay Kit (Pierce) using bovine serum albumin as standard.

### Measurement of fungal growth

Overnight cultures of *C*. *albicans* were diluted in 200 μL of fresh YPD medium (starting OD_600_: 0.1) in 96-well microtiter plates. Fungal growth was monitored spectrophotometrically at OD_600_ at 1-h intervals (up to 24 h) at 30°C.

### Examination of yeast-to-hyphal morphological transition

The ability of the *C*. *albicans* orf19.3727 null mutants to form germ tube was evaluated by incubating fungal cells (250 μL, 10^5^ cells/mL) with fetal calf serum at 37°C for 2 h [21]. A germ tube was defined as blastospore with a slender, cylindrical apical extension at least one blastospore diameter in length. Clumped cells were excluded. Three hundred cells were examined and the percentage of germ tube-forming cells was calculated.

### Adhesion to buccal epithelial cells (BECs)

Human BECs were collected from 16 healthy adults (eight males, eight females) by gently rubbing the cheek mucosa with sterile swabs [21]. The BECs were washed twice in phosphate-buffered saline (PBS). Equal volume of BECs (10^5^ cells/mL) and fungal cells (10^7^ cells/mL) was incubated at 37°C for 1 h with agitation (75 rpm), and filtered through 12 μm pore polycarbonate filters. Unattached cells were removed by washing, and the BECs were air-dried and Gram’s stained. The number of fungal cells attached to uniform BECs was counted under light microscope in 50 BECs.

### Virulence study using reconstituted human epithelial (RHE) tissues

Semisynchronized *C*. *albicans* for infection study was prepared as described [22]. For infection study, fungal cells (2 × 10^6^ cells in 50 μL of PBS) were added to oral (RHO/S/5) reconstituted human epithelial (RHE) tissues (0.5 cm^2^, SkinEthic Laboratories) and incubated at 37°C, 5% CO_2_, 100% humidity for 48 h. Uninoculated controls (PBS only) were included for comparison.

To observe histological changes upon tissue invasion by *C*. *albicans*, the RHE tissues were dissected in half. Sections of 1 μm-thick were cut from the base of the midline towards the outer margin, stained with hematoxylin and eosin, and viewed under light microscope at ×200 magnification. The released lactate dehydrogenase (LDH) from the RHE tissues upon fungal invasion was determined using CytoTox 96 nonradioactive cytotoxicity assay (Promega) [23].

### Statistical analysis

All experiments were carried out in three separate occasions, and triplicate was performed in each occasion. All data were expressed as the mean values with the corresponding standard deviations (S.D.). Statistical significance between treated and control groups was assessed by Student's *t* test. A *p*-value < 0.05 was considered statistically significant.

### Ethics statement

The present study involved human tissue samples, and an approval was obtained from the Human Subjects Ethics Sub-committee of the Technological and Higher Education Institute of Hong Kong. Each volunteer’s written consent was obtained prior to the recruitment into the present study.

## Results and discussion

This study was a continuation of our research on the functional significance of phytase activity in the ubiquitous human fungal pathogen *Candida*. Our previous study revealed the presence of cell-associated phytase activity in *Candida* [15], and here, we further investigated the functional role of phytase activity in *C*. *albicans* pathobiology.

**Fig 1:**
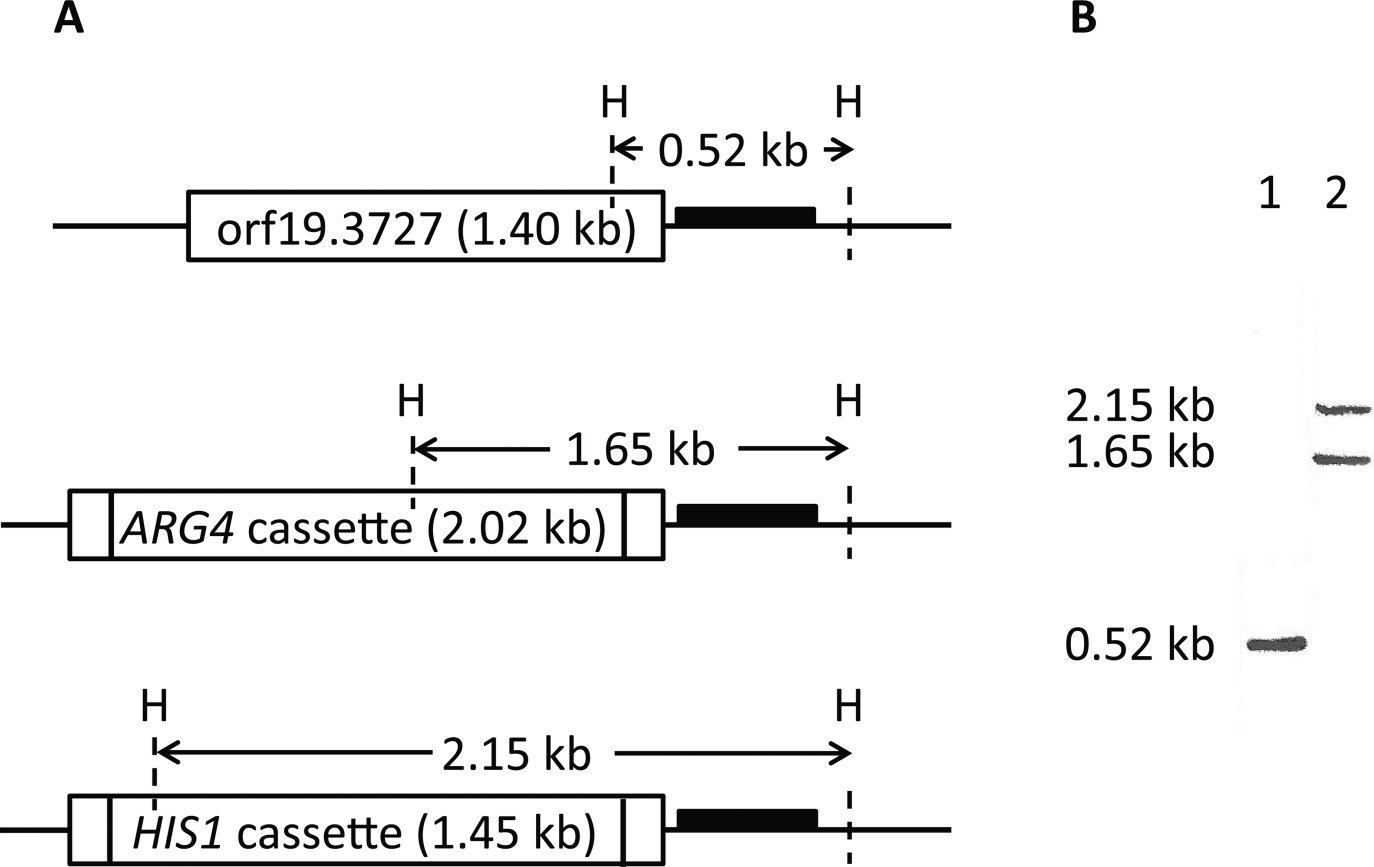
Disruption of orf19.3727 in *C*. *albicans*. (A) Schematic diagrams of intact chromosomal *C*. *albicans* orf19.3727 (top), *C*. *albicans* orf19.3727 disrupted by *ARG4* cassette (middle), and *C*. *albicans* orf19.3727 disrupted by *HIS1* cassette (bottom). Genomic DNA was isolated by glass bead method and restricted by *Hin*cII. A 268-bp PCR product (black bar) was used as a probe. (B) A representative Southern hybridization: lane 1: *C*. *albicans* BWP17; lane 2: *C*. *albicans* orf19.3727 null mutant.

A PCR-based gene targeting method [17] was employed to disrupt both chromosomal copies of *C*. *albicans* orf19.3727. The resultant *C*. *albicans* orf19.3727 null mutants were confirmed by PCR and genomic Southern hybridization (Fig 1). We also generated corresponding reintegrants by inserting a full length gene sequence spanning from -1 kb to +1 kb of *C*. *albicans* orf19.3727 into the null mutants for comparison.

**Fig 2:**
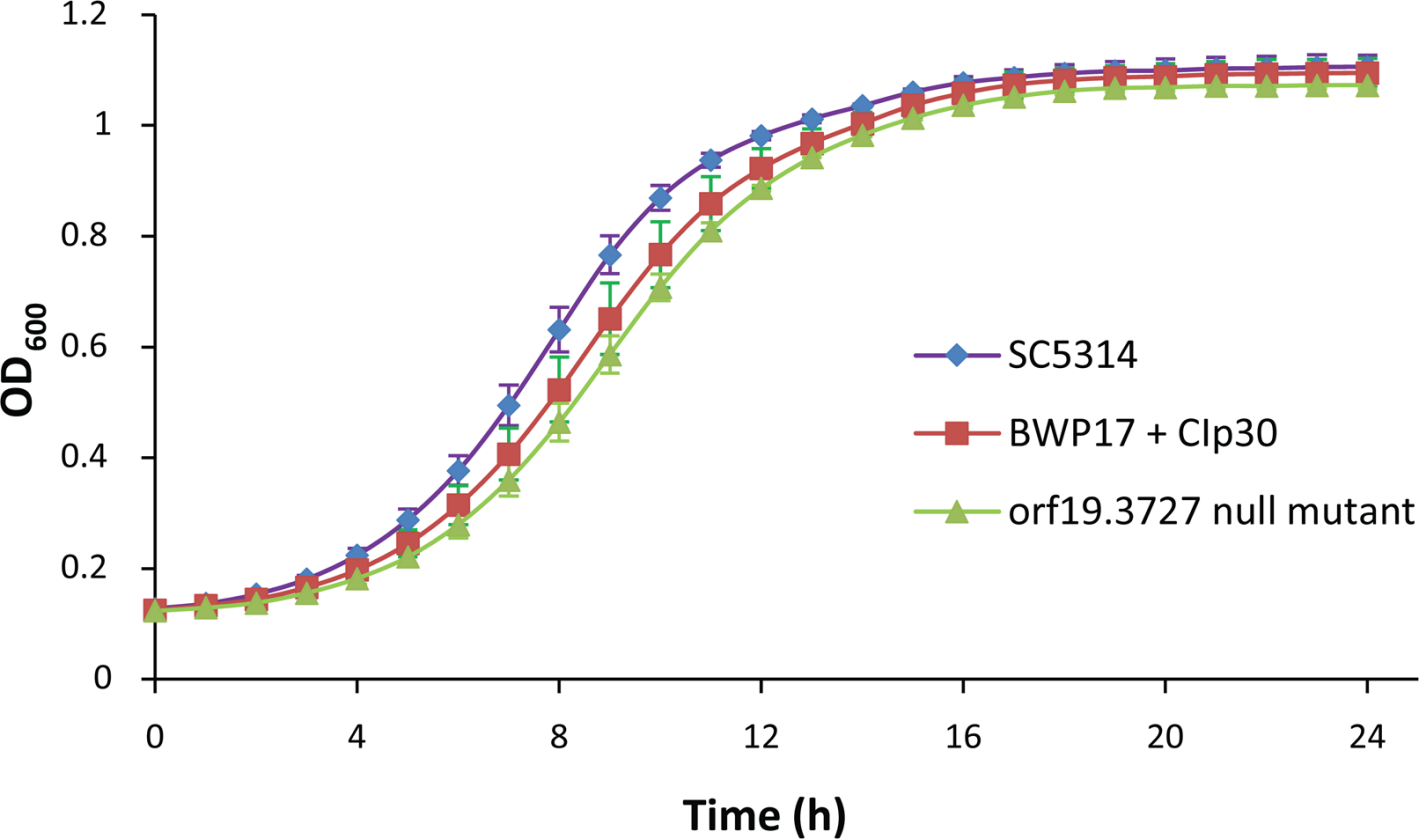
Comparison of the growth rates of *C*. *albicans* strains. Fungal cells were grown in YPD medium at 30°C. Aliquots were withdrawn at 1-h intervals for a period of 24 h.

The cellular phytase activity of the *C*. *albicans* orf19.3727 null mutants and the reintegrants were then compared. There was a reduction of ~25% of cell-associated phytase activity in the *C*. *albicans* orf19.3727 null mutants (1.03 ± 0.09 U/mg; *p* < 0.05) and such phytase activity was rescued in the reintegrants (1.40 ± 0.15 U/mg), suggesting that orf19.3727 functions in phytase activity in *C*. *albicans*. The phytase activities in wild type strain *C*. *albicans* SC5314 and *C*. *albicans* BWP17 + CIp30 were 1.39 ± 0.11 U/mg and 1.41 ± 0.08 U/mg respectively. Up to now, there has been no authentic phytase reported in *C*. *albicans*. The residual phytase activity in *C*. *albicans* orf19.3727 null mutants (~75%) might be contributed by other cytosolic or cellular enzymes such as acid phosphatase that also degraded the substrate phytate. However, orf19.3727 is not an essential gene in *C*. *albicans* for core cellular metabolism for survival and proliferation as the growth rates of the *C*. *albicans* orf19.3727 null mutants and the reintegrants were similar to that of the wild type strain *C*. *albicans* SC5314 and *C*. *albicans* BWP17 + CIp30 (Fig 2).

A clinically relevant virulence trait of *C*. *albicans* is its ability to exist in different morphological forms (budded yeast, pseudohyphae, and true hyphae). Inhibition of this yeast-to-hyphal morphological transition has been regarded as a prime antifungal strategy [24]. To understand the correlates between phytase activity and morphogenesis in *C*. *albicans*, we first estimated the number of hyphal cells in the *C*. *albicans* orf19.3727 null mutants under hyphal-inducing conditions at 37°C, in the presence of fetal calf serum which triggers hyphal growth. Hyphal growth was reduced by ~22% in the *C*. *albicans* orf19.3727 null mutants (75.16 ± 3.25%; *p* < 0.05) compared with the reintegrants (96.55 ± 2.01%). The respective data of the wild type strain *C*. *albicans* SC5314 and *C*. *albicans* BWP17 + CIp30 were 96.17 ± 1.47% and 97.66 ± 1.59%. These data suggest a dual role of *C*. *albicans* orf19.3727 in both phytase activity as well as in fungal morphogenesis. Further studies are however necessary to elucidate the key player(s) in cellular circuitry and signaling pathways linking between these two cellular phenomena.

Furthermore, adhesion to mucosal epithelial cells is a critical prerequisite for successful fungal invasion and is a recognized virulence attribute of *C*. *albicans* [21]. Therefore, we determined the ability of the *C*. *albicans* orf19.3727 null mutants to adhere to BECs. The numbers of *C*. *albicans* orf19.3727 null mutants attached to BECs was reduced by ~22% (99 ± 3; *p* < 0.05), and yet this attribute reverted in the reintegrants (135 ± 5) which was similar to that of the wild type strain *C*. *albicans* SC5314 (128 ± 5) and *C*. *albicans* BWP17 + CIp30 (133 ± 6).

**Fig 3:**
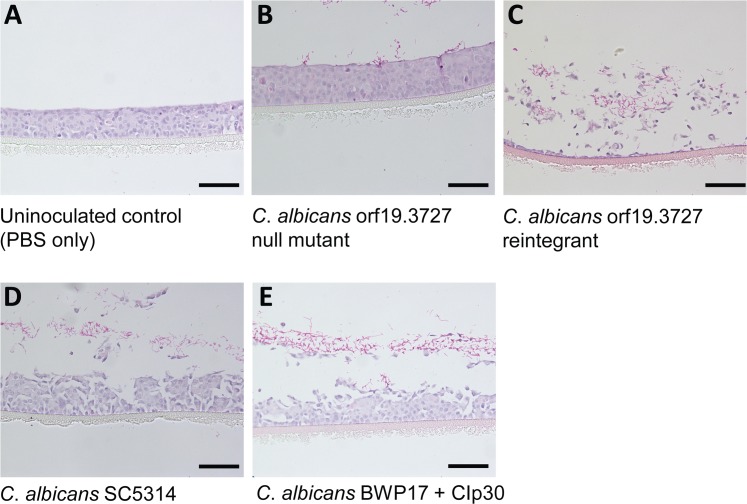
Determination of the virulence of *C*. *albicans* strains using reconstituted human epithelial (RHE) tissues. *Candida* cells were inoculated with RHE tissues and incubated at 37°C, 5% CO_2_, 100% humidity for 48 h and histopathological sections were then obtained and stained with hematoxylin (purple) and eosin (pink) and viewed under light microscope at ×200 magnification. (A) Uninoculated control (PBS only). (B) *C*. *albicans* orf19.3727 null mutant. (C) *C*. *albicans* orf19.3727 reintegrant. (D) *C*. *albicans* SC5314. (E) *C*. *albicans* BWP17 + CIp30. Note that hyphal invasion (pink) and destruction of the RHE cellular architecture in C, D, and E but not in the uninoculated PBS control A, and the null mutant B where all cell layers are intact. Scale bar = 100 μm.

**Fig 4:**
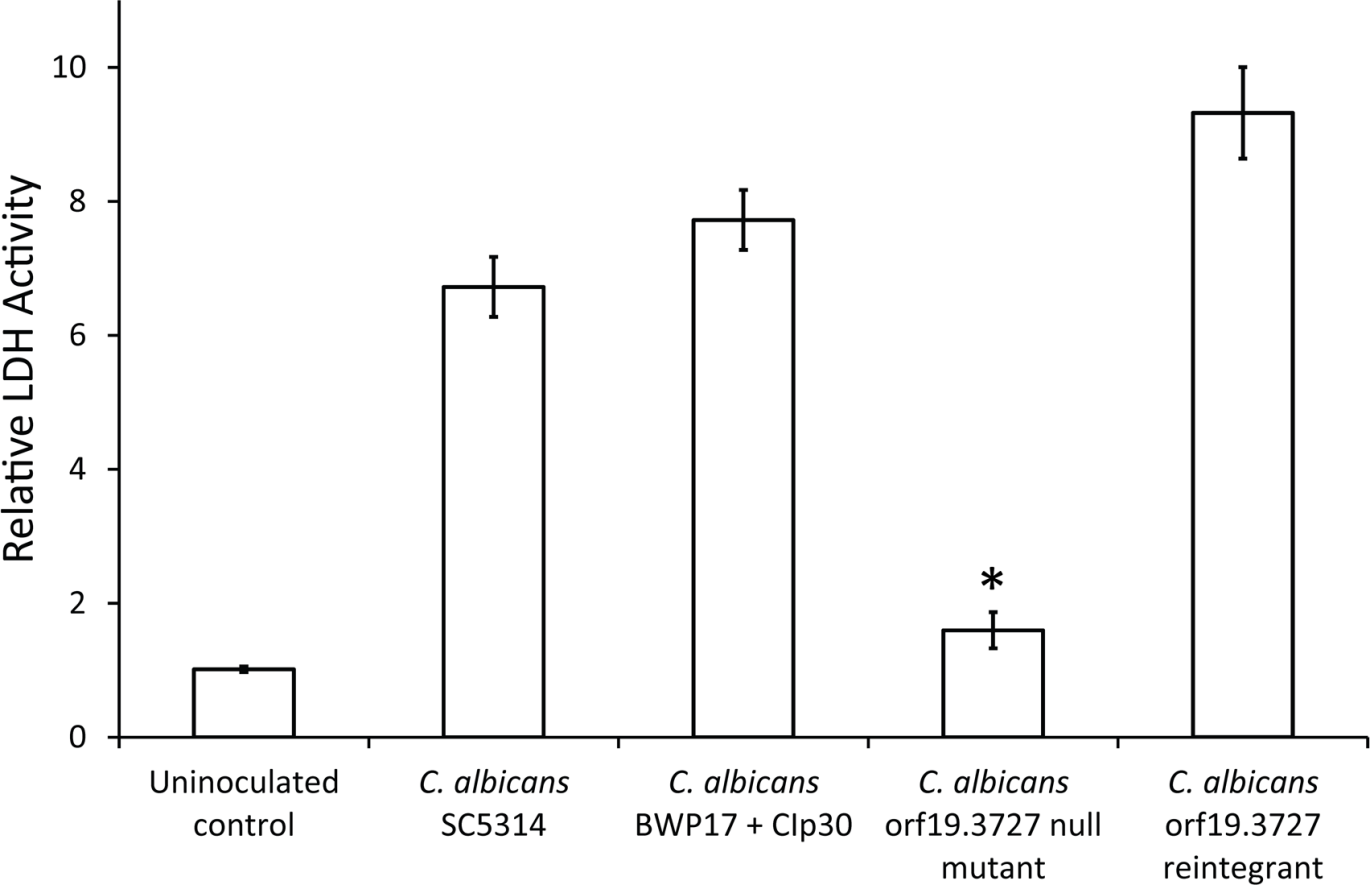
Determination of lactate dehydrogenase (LDH) from the culture supernatants of the RHE tissues upon fungal invasion. The amount of released LDH was determined using CytoTox 96 nonradioactive cytotoxicity assay (n = 3; *p < 0.05).

As *Candida* phytase is a cell surface protein, we speculated that phytase might play a key functional role in cell-cell interaction, a prerequisite for fungal penetration and invasion of host tissues. To this end, we employed the well-established three-dimensional *in vitro* models of RHE tissues to evaluate the contribution of phytase activity to pathogenicity. The RHE tissues histologically closely resemble the human mucosa, and can be cultivated in conditions that closely imitate the physiological habitat where all major biomarkers for *in vivo* cell repair are expressed. These *in vitro* models of candidiasis, which mimic *in vivo* condition, have been used extensively to study *Candida* virulence [22,23]. The *C*. *albicans* orf19.3727 null mutants were allowed to infect the RHE tissues and a reduced level of tissue damage was evident, whereas full capacity of tissue damage could be rescued in the reintegrants and the two reference strains (Fig 3). The respective degree of tissue damage was also determined quantitatively by measuring the amount of released LDH. The ability of the *C*. *albicans* orf19.3727 null mutants to infect oral RHE tissues was significantly reduced by ~75%, whereas the tissue damage caused by the reintegrants was similar to that of the wild type strain *C*. *albicans* SC5314 and *C*. *albicans* BWP17 + CIp30 (Fig 4). Our findings suggested a strong correlation between *C*. *albicans* orf19.3727 and *in vitro* fungal invasion, a primary step in systemic candidiasis.

## Conclusions

To conclude, through the study of the *C*. *albicans* orf19.3727 null mutants, we have demonstrated a close correlation between *C*. *albicans* phytase activity, morphogenesis, and pathogenesis, and this opens up an exciting research landscape that focuses on a novel approach to strategic antifungal drug designs. In accordance with the *Candida* genome database (http://www.candidagenome.org), two other orfs (i.e. orf19.2619 and orf19.11238) are very similar (>96%) to orf19.3727 that could contribute to phytase activity. However, they are merged together in Assembly 20 and denote the same gene locus as orf19.3727, suggesting that these orfs are “alias”. One aspect for further study could be to utilize fluorescent molecules (e.g. GFP) tagged *C*. *albicans* orf19.3727 strains to characterize small molecules that downregulate phytase activity. Another could be the delineation of additional cellular parameters impacting *C*. *albicans* orf19.3727 and virulence traits by transcript profiling using microarray. In translational terms, our data imply that suppression of phytase activity of this common human fungal pathogen responsible for recalcitrant invasive candidiasis could be another avenue that could be exploited to suppress its major virulence attributes and thus reduce the overall mycotic disease burden worldwide.
